# Galectin-3 and the Glyco-Inflammatory Axis: A Missing Link to Residual Cardiovascular Risk in Coronary Artery Disease

**DOI:** 10.3390/biomedicines14010021

**Published:** 2025-12-22

**Authors:** Toshiki Otoda, Ken-ichi Aihara, Ken-ichi Matsuoka, Tadateru Takayama

**Affiliations:** 1 Division of General Medicine, Department of Internal Medicine, Nihon University School of Medicine, 30-1, Oyaguchi Kami-cho, Itabashi-ku, Tokyo 173-8610, Japan; takayama.tadateru@nihon-u.ac.jp; 2 Department of Community Medicine and Medical Science, Graduate School of Biomedical Sciences, Tokushima University, 3-18-15 Kuramoto-cho, Tokushima 770-8503, Japan; aihara@tokushima-u.ac.jp; 3 Department of Hematology, Endocrinology and Metabolism, Graduate School of Biomedical Sciences, Tokushima University, 3-18-15 Kuramoto-cho, Tokushima 770-8503, Japan; k-matsu@tokushima-u.ac.jp

**Keywords:** coronary artery disease, residual cardiovascular risk, Galectin-3, glycosylation, lectins, inflammation, fibrosis, lysosomal stress, biomarker, therapeutic target

## Abstract

Residual cardiovascular risk remains a major challenge in coronary artery disease, even after optimal lipid-lowering and anti-inflammatory therapy. Beyond classical risk factors, persistent low-grade inflammation and fibrotic remodeling contribute to adverse outcomes that current treatments fail to fully prevent. Growing evidence highlights the glyco-inflammatory axis—the interplay between protein glycosylation-dependent signaling and inflammation—as an underappreciated contributor to residual atherosclerotic risk, largely because current therapeutic strategies do not directly target glycan-mediated mechanisms. Within this framework, Galectin-3 (Gal-3), a β-galactoside-binding lectin, has emerged as a key molecular hub linking metabolic stress, lysosomal dysfunction, and vascular remodeling. By recognizing specific glycan motifs on immune and stromal cells, Gal-3 orchestrates macrophage activation, endothelial dysfunction, and extracellular matrix deposition, thereby amplifying chronic inflammation and fibrosis. Elevated circulating Gal-3 levels are associated with plaque vulnerability and major adverse cardiovascular events, independent of lipid or C-reactive protein levels. Experimental Gal-3 inhibition reduces inflammation and fibrosis in preclinical models, supporting its therapeutic potential. This review integrates mechanistic, translational, and clinical evidence to propose Gal-3 as a missing link between intracellular stress responses and extracellular fibro-inflammatory remodeling. Targeting the Gal-3-mediated glyco-inflammatory axis may represent a novel strategy to overcome residual cardiovascular risk and achieve comprehensive vascular protection in the post-statin era.

## 1. Introduction

Coronary artery disease (CAD) remains the leading cause of morbidity and mortality worldwide despite significant lipid-lowering, antithrombotic, and anti-inflammatory therapy advances [1,2]. Even under optimal statin therapy and well-controlled low-density lipoprotein cholesterol (LDL-C) levels, a substantial proportion of patients continue to experience adverse cardiovascular events—a phenomenon termed residual risk [3]. Persistent vascular inflammation has been recognized as a mechanistically distinct contributor to this residual risk, independent of LDL-C lowering [4]. Epidemiological evidence further demonstrates that systemic inflammatory markers remain strong predictors of cardiovascular events in secondary prevention cohorts, even when lipid parameters are fully optimized [5]. Recent consensus emphasizes that residual inflammatory risk is not merely a prognostic marker but represents a modifiable therapeutic domain in contemporary CAD management [6]. Additionally, this concept is supported in chronic coronary syndrome populations, where continuous low-grade inflammation drives progressive vascular injury despite standard-of-care lipid-lowering therapy [7].

Besides statins, long-term PCSK9 inhibitor outcome data confirm that even when LDL-C levels are reduced to ultralow ranges, cardiovascular events are not completely eliminated, highlighting persistent immune activation as a key determinant of residual cardiovascular risk [8,9]. Similarly, clinical intravascular ultrasound studies demonstrate that plaque regression under intensive lipid-lowering is attenuated in patients with impaired glucose metabolism [10,11]. Furthermore, carotid ultrasound imaging has demonstrated that echolucent, inflammation-rich plaques and elevated high-sensitivity C-reactive protein (CRP) continue to predict cardiovascular events in patients with well-controlled LDL-C [12]. Therefore, residual risk arises from both cholesterol-mediated mechanisms and enduring vascular inflammation, fibrotic remodeling, oxidative stress, and metabolic dysregulation within the arterial wall [2,13,14,15]. Importantly, therapeutic strategies focusing solely on lipid metabolism fail to address the broader biological drivers of ongoing vascular injury [1,2,16].

These clinical findings emphasize the importance of inflammation-driven mechanisms—including those mediated by Galectin-3 (Gal-3)—in residual cardiovascular risk pathogenesis. Elevated Gal-3 levels have been associated with higher event rates in patients with CAD even when lipid parameters are well controlled; thus, Gal-3 may contribute to vascular immune activation beyond cholesterol-dependent mechanisms [13,17,18].

Emerging evidence suggests that post-translational modifications, particularly glycosylation, and their recognition by lectins represent a crucial yet underexplored regulatory layer in cardiovascular disease [19,20,21,22,23]. The glycan–lectin network orchestrates diverse biological processes, including immune cell activation, cell adhesion, extracellular matrix organization, and fibrosis—all of which are central to atherosclerosis initiation and progression [19,20,21,22,24,25]. Aberrant glycosylation patterns on endothelial and immune cell surface proteins alter receptor–ligand interactions and inflammatory signaling, contributing to vascular dysfunction [26,27,28]. In this context, glycan-binding proteins such as galectins, selectins, and siglecs are emerging as key modulators linking metabolic cues to immune responses [19,20,21,24].

Among these, Gal-3 has attracted considerable attention as a pleiotropic mediator at the intersection of inflammation, fibrosis, and metabolism [18,25,29,30,31,32]. As a β-galactoside-binding lectin expressed in macrophages, endothelial cells, and fibroblasts, Gal-3 modulates multiple steps in atherogenesis—from foam cell formation and macrophage activation to extracellular matrix deposition and plaque stability [32,33,34,35]. Gal-3 is upregulated under lysosomal membrane permeabilization conditions, amplifying sterile inflammation and fibrotic signaling responses [25,36,37,38,39,40]. Additionally, elevated circulating Gal-3 has been linked to adverse outcomes in patients with CAD, heart failure, and metabolic disorders, suggesting its role as both a biomarker and a residual cardiovascular risk pathogenic effector [13,14,17,18,41,42,43].

Although several studies have demonstrated the prognostic and diagnostic value of Gal-3 in cardiovascular disease, its causal involvement in atherogenesis remains incompletely understood [13,14,17,18,29,41,44]. Mendelian randomization studies have not yet confirmed a direct causal link; additionally, clinical investigations have often approached Gal-3 primarily as a biomarker rather than an active disease driver [14,43,45]. Furthermore, only a few reports have contextualized Gal-3 within the glyco-lectin axis, a conceptual framework integrating aberrant glycosylation, immune activation, and fibrotic progression in CAD [20,24,44].

These modifications create high-affinity glycan ligands for Gal-3. Gal-3 specifically recognizes extended poly-LacNAc structures but also tolerates 3′-sialylated and 2′-fucosylated LacNAc motifs [46,47]. Importantly, Gal-3 binds strongly to ABH blood-group–related glycans displayed onvascular endothelial cells, where α1-2 fucosylation enhances Gal-3 affinity [47].

**Figure 1:**
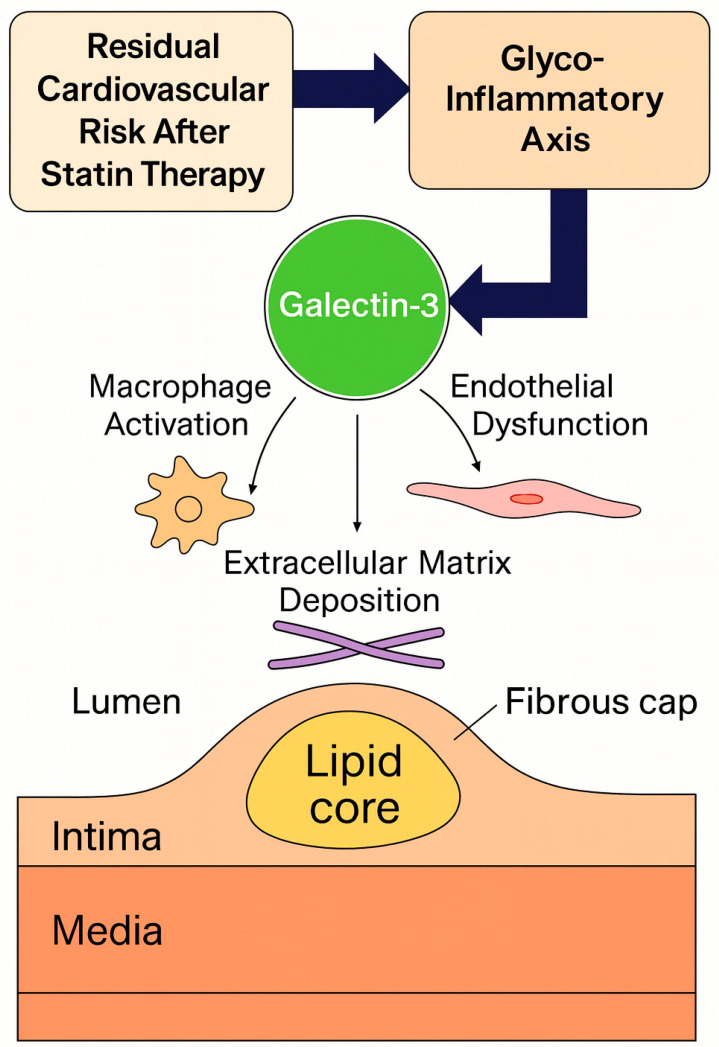
Conceptual framework of the glyco-inflammatory axis linking glycosylation, inflammation, and fibrosis in coronary artery disease.

Therefore, this review provides an integrated overview of the glyco-inflammatory axis in CAD, focusing on Gal-3 as a mechanistic link connecting lysosomal stress, inflammation, and extracellular matrix remodeling (Figure 1). By elucidating the biological and clinical significance of Gal-3 in residual cardiovascular risk, we highlight its potential as a novel diagnostic and therapeutic target in the era of precision cardiometabolic medicine.

## 2. The Glyco-Inflammatory Axis in Cardiovascular Disease

### 2.1. Definition and Conceptual Framework

The cardiovascular system is tightly regulated by molecular networks integrating metabolic, inflammatory, and structural cues. Among these, the glyco-inflammatory axis—defined as the dynamic interplay between protein glycosylation and inflammation—has recently emerged as a fundamental mechanism in vascular biology [19,20]. Glycosylation is among the most abundant and diverse post-translational modifications, influencing the folding, stability, localization, and function of numerous membrane and secreted proteins [19,21]. Importantly, glycan structures represent molecular “barcodes” recognized by glycan-binding proteins (lectins), thereby modulating cell–cell and cell–matrix interactions within the vessel wall [19,20,21,22]. Inflammatory signaling reshapes the cellular glycome, increasing β1,6-GlcNAc branching and extending poly-N-acetyllactosamine (LacNAc) chains—structures preferentially recognized by Gal-3. Thus, the glyco-inflammatory axis is not simply an association between inflammation and glycosylation, but a feed-forward system whereby glycan remodeling enhances lectin-mediated immune activation, positioning Gal-3 as a central molecular amplifier in atherosclerosis.

### 2.2. Inflammatory Reprogramming and Alterations in Glycosylation

In atherosclerosis, aberrant glycosylation profoundly affects endothelial and immune cell behavior [19,20,21,22]. Proinflammatory cytokines such as tumor necrosis factor-α (TNF-α) and interleukin-1β can reprogram the activity of glycosyltransferases, causing altered N- and O-glycan branching on adhesion molecules and cytokine receptors [20,22,26]. These changes enhance leukocyte adhesion, monocyte recruitment, and macrophage retention within the intima [20,22,26], promoting immune cell retention within plaques. Importantly, chronic inflammatory signaling does not merely increase total glycan abundance—it qualitatively remodels the vascular glycome. Upregulation of N-acetylglucosaminyltransferase V (MGAT5) drives β1,6-GlcNAc branching and elongation of poly–N-acetyllactosamine (poly-LacNAc) chains, while downregulation of sialyltransferases (e.g., ST6GAL1) reduces terminal sialylation. Concurrently, increased fucosyltransferase activity (FUT4/FUT7) enhances α1-2 fucosylation, particularly within ABH-type blood group antigens.

These modifications expose multivalent LacNAc-rich motifs that markedly increase affinity for Gal-3, enabling high-avidity binding and oligomerization. The resulting glycan landscape forms an optimized platform for Gal-3 lattice formation, prolonging receptor surface residency and potentiating downstream inflammatory and fibrotic signaling. Consequently, inflammation-driven glycome remodeling acts as a molecular primer that facilitates Gal-3 engagement, linking metabolic stress to sustained plaque progression.

Furthermore, oxidative stress and hyperglycemia—hallmarks of metabolic syndrome and diabetes—exacerbate this process by disturbing glycan biosynthesis and promoting sialylated and fucosylated epitope expression, thereby further amplifying chronic inflammation and fibrotic signaling [19,20,21,22,28].

### 2.3. Lectin Family Functional Roles

Lectins interpret cell-surface glycans as biochemical “codes” and thereby regulate leukocyte trafficking, immune tolerance, and vascular homeostasis [19,21,26,27]. Among them, selectins (E-, P-, and L-selectin) recognize sialylated and fucosylated glycans and mediate the initial rolling and tethering of leukocytes on activated endothelium, while sialic acid—binding immunoglobulin-like lectins (Siglecs) mostly act as inhibitory receptors that dampen immune activation by recognizing sialylated self-glycans and transmitting ITIM-based signals [20,21]. These families are important for cardiovascular immunobiology but primarily function at the level of leukocyte recruitment and immune “braking.”

In contrast, galectins—particularly Gal-3—operate at the interface of inflammation and tissue remodeling. Galectins are β-galactoside-binding lectins that recognize N-acetyllactosamine (LacNAc) motifs on N- and O-glycans [29,30].

Gal-1 is a prototype galectin with immunoregulatory properties, but Gal-3 is unique as the only chimera-type galectin, containing an N-terminal oligomerization domain and a C-terminal carbohydrate-recognition domain (CRD). This structural organization allows Gal-3 to form multimers and cross-link multiple LacNAc-rich glycoproteins simultaneously, creating higher-order “lattices” on the cell surface.

Beyond Galectin-1 and Gal-3, other galectin isoforms also modulate cardiovascular inflammation. Galectin-8 has been reported to function as a matricellular regulator of integrin-mediated adhesion and endothelial activation under oxidative or mechanical stress, suggesting a potential role in early atherogenic signaling [48]. Galectin-9 regulates T-cell and macrophage polarization and, in preclinical cardiovascular models, has been shown to attenuate chronic vascular inflammation [49]. Although less extensively studied than Gal-3, these isoforms underscore the broader relevance of the galectin family in cardiovascular immunobiology and further justify the present focus on Gal-3, the isoform most consistently linked to glyco-inflammatory remodeling in CAD.

Through this glycan-dependent lattice formation, Gal-3 stabilizes and clusters glycosylated receptors such as integrins, growth factor receptors, and cytokine receptors on endothelial cells, macrophages, and fibroblasts [29,30]. Receptor clustering prolongs their surface residency, delays endocytosis, and sustains downstream signaling, thereby amplifying proinflammatory, pro-fibrotic, and pro-oxidative pathways within the vascular wall. Unlike Siglecs, which generally act as negative regulators of immune activation, Gal-3 functions as a context-dependent amplifier of injury signals, particularly when the glycome has been remodeled toward poly-LacNAc-rich, Gal-3-favored structures as described above.

Consequently, within the broader lectin family, Gal-3 occupies a distinctive position as a “glyco-sensor and effector” that converts inflammation-induced changes in glycosylation into persistent cellular activation and matrix remodeling in cardiovascular disease. This mechanistic role makes Gal-3 a central node of the glyco-inflammatory axis and provides the rationale for focusing on Gal-3 in the subsequent sections of this review.

### 2.4. Relationship with Residual Risk in CAD

In coronary artery disease, persistent activation of this glyco-inflammatory axis contributes to residual risk beyond lipid control [1,13,18,41,50]. Specifically, dysregulated glycosylation enhances endothelial dysfunction, macrophage activation, and extracellular matrix remodeling—key drivers of plaque progression and instability [13,18,19,20,21,22,29,41]. Particularly, Gal-3 binds to glycosylated receptors such as integrins, laminins, and transforming growth factor-β (TGF-β) receptors, potentiating fibrotic and proinflammatory signaling [13,14,18,30,41]. However, Gal-3 does not bind indiscriminately—it preferentially recognizes poly-N-acetyllactosamine-extended (poly-LacNAc) N-glycans, particularly when β1,6-GlcNAc branching is increased under inflammatory conditions. This high-avidity interaction enables Gal-3 to multimerize via its N-terminal domain, forming glycan lattices that cluster integrins, TGF-β receptors, and other glycoproteins on the cell surface. Such lattice formation delays receptor endocytosis, stabilizes signaling platforms, and sustains downstream activation of SMAD, NF-κB, and fibro-inflammatory pathways. Through this mechanism, Gal-3 effectively converts transient inflammatory stimuli into chronic vascular remodeling, providing a molecular explanation for persistent residual risk even after LDL-lowering or anti-inflammatory therapy

Taken together, glyco-inflammatory network perturbation represents both a hallmark of advanced atherosclerosis and a potential therapeutic target for mitigating residual inflammatory and fibrotic risk [1,14,30,50].

This concept is further supported by intravascular ultrasound research demonstrating that plaque regression during statin therapy is attenuated in patients with poor glycemic control, suggesting that metabolic–inflammatory stress contributes substantially to residual risk [11].

This conceptual framework provides a molecular basis to understand why conventional lipid-lowering or anti-inflammatory therapies may incompletely suppress cardiovascular risk. By focusing on the glycan–lectin interface, new opportunities arise for biomarker discovery, pathway-specific intervention, and precision risk stratification in patients with CAD.

In the next section, we detail the glycan ligands of Gal-3 and how this glycan-dependent receptor clustering directly drives atherosclerotic progression.

## 3. Gal-3 Biology and Pathophysiological Roles

Although multiple galectin family members participate in immune regulation, including Galectin-1, -8, and -9, Gal-3 exhibits a uniquely potent combination of β-galactoside-binding specificity and N-terminal-mediated oligomerization that enables stable lattice formation. Unlike prototype galectins such as Gal-1, which often exert immunoregulatory or tolerogenic functions, Gal-3 predominantly amplifies inflammatory and fibrotic pathways in cardiovascular tissue. This structural and functional specialization explains why Gal-3, among the galectin family, has emerged as the principal glyco-inflammatory mediator implicated in CAD. Gal-3 is a chimera-type β-galactoside-binding lectin encoded by the *LGALS3* gene, uniquely combining a C-terminal carbohydrate recognition domain with an N-terminal collagen-like domain that enables oligomerization. This modular structure allows Gal-3 to cross-link glycosylated receptors and extracellular matrix (ECM) components, forming multivalent lattices that regulate cell signaling, adhesion, and tissue remodeling [24,30,51,52].

Gal-3 is also a prototypical “matricellular” protein—an extracellular mediator that modulates cell–matrix interactions without serving a structural role in the matrix itself. This places Gal-3 alongside proteins such as osteopontin and thrombospondins, which act as contextual amplifiers of stress and inflammation within the vascular microenvironment. Like other members of the galectin family, Galectin-3 lacks a signal peptide and is secreted via non-classical pathways rather than the conventional endoplasmic reticulum–Golgi route [53,54]. However, Galectin-3 is distinctive in that its extracellular release is tightly linked to lysosomal membrane damage and cellular stress. Upon lysosomal membrane permeabilization, Galectin-3 accumulates in the cytosol and can be released extracellularly through non-classical exocytosis or passive leakage from damaged cells, thereby coupling intracellular danger sensing with paracrine inflammatory signaling.

### 3.1. Cellular Localization and Regulation

Gal-3 is widely expressed in macrophages, monocytes, endothelial cells, vascular smooth-muscle cells (VSMCs), and fibroblasts. In the cytoplasm, it associates with mitochondria and lysosomes, where it contributes to organelle integrity and autophagic flux [55,56]. Under metabolic or oxidative stress, lysosomal membrane permeabilization (LMP) causes Gal-3 to translocate to the cytosol, recognizing exposed β-galactoside residues on ruptured endolysosomal membranes [40,55,57]. This process represents an intracellular danger signal that triggers inflammatory cascades, including NLRP3 inflammasome activation and proinflammatory cytokine release [56,58]. Persistent or excessive LMP causes extracellular Gal-3 secretion, converting a cell-intrinsic stress response into a paracrine inflammatory cue. In metabolic disorders such as obesity and type 2 diabetes, chronic nutrient overload and lipotoxicity further enhance macrophage activation and lysosomal stress, driving sustained Gal-3 release into the extracellular space. Thus, this metabolic–inflammatory milieu positions Gal-3 as a molecular interface linking metabolic dysregulation with innate immune activation and subsequent fibro-inflammatory remodeling across organs [59].

### 3.2. Functional Roles in Inflammation and Fibrosis

Extracellular Gal-3 binds to glycosylated receptors such as integrins, CD98, and the TGF-β receptor complex, thereby amplifying downstream profibrotic signaling [30,31,32]. Importantly, Gal-3 does not bind all glycoconjugates equally, but shows high affinity for β-galactoside-containing N-glycans enriched in poly-N-acetyllactosamine (poly-LacNAc) repeats. Inflammatory signaling upregulates MGAT5-driven β1,6-GlcNAc branching and reduces terminal sialylation, exposing LacNAc motifs that favor Gal-3 engagement. Increased α1-2 fucosylation—particularly within ABH-type blood group antigens—further enhances multivalent binding, enabling Gal-3 to form stable receptor clusters on endothelial cells and macrophages. These glycan architectures are therefore not passive markers but active determinants of Gal-3-mediated signaling strength.

Through multivalent binding to poly-LacNAc-rich N-glycans, Gal-3 undergoes N-terminal oligomerization, enabling the formation of supramolecular “lattices” that cluster cell-surface receptors into stable signaling platforms. This lattice assembly prevents rapid receptor endocytosis, prolongs their membrane residency, and sustains downstream activation of SMAD, NF-κB, MAPK, and JAK–STAT pathways. As a result, transient inflammatory cues are converted into long-lasting fibro-inflammatory responses. In endothelial cells and macrophages, Gal-3-mediated lattice formation enhances integrin signaling, promotes focal adhesion stability, and reinforces chemokine and cytokine production, thereby driving persistent vascular inflammation.

Major cell-surface targets of Gal-3 carry distinct glycan signatures that enable high-avidity binding and lattice formation. Integrins (particularly β1 and αvβ3) possess MGAT5-dependent β1,6-GlcNAc-branched N-glycans with extended poly-LacNAc repeats, providing an optimal scaffold for Gal-3 cross-linking. TGF-β receptor II similarly displays LacNAc-rich N-glycans that, when clustered by Gal-3, enhance SMAD phosphorylation and drive fibroblast-to-myofibroblast differentiation. Endothelial adhesion molecules such as CD146 and CD98 harbor partially desialylated and α1-2-fucosylated glycans that further increase Gal-3 affinity. Importantly, the actual Gal-3 ligands within these glycoproteins are the poly-LacNAc-extended, β1,6-branched N-glycans themselves. In integrins, these MGAT5-dependent N-glycans constitute the principal Gal-3 binding sites, whereas TGF-β receptors present LacNAc-rich N-glycans that specifically function as high-avidity Gal-3 ligands.

ECM proteins, including laminin and fibronectin, contain LacNAc-bearing glycans that can serve as secondary scaffolds for Gal-3 oligomerization, thereby reinforcing matrix stiffness and collagen deposition. Together, these ligand-specific interactions highlight that Gal-3 function is determined not simply by receptor abundance but by the inflammatory remodeling of the vascular glycome.

Building upon these lattice-mediated interactions, Gal-3 further promotes monocyte adhesion, macrophage activation, and fibroblast proliferation [30,31,32,60]. Within the vascular microenvironment, it promotes macrophage transition to a proinflammatory phenotype and stimulates VSMC migration and collagen deposition, linking inflammation to structural remodeling [32,60]. Moreover, Gal-3 interacts with advanced glycation end-products and oxidized lipoproteins, further reinforcing oxidative and metabolic stress responses characteristic of diabetes-associated atherosclerosis [30,61].

### 3.3. Clinical and Translational Implications

Circulating Gal-3 concentrations correlate with disease severity and adverse outcomes across cardiovascular and metabolic disorders [13,14,18,30,41]. Elevated serum levels have been reported in acute coronary syndrome (ACS), chronic CAD, and heart failure, reflecting both ongoing inflammation and fibrotic activity [13,14,18,30,41]. These observations suggest a broad association between Gal-3 and cardiometabolic pathology; however, interpretation of circulating Gal-3 is complicated by its partial dependence on renal clearance. A well-recognized challenge in interpreting circulating Gal-3 is its partial dependence on renal clearance. Serum Gal-3 levels rise in parallel with declining eGFR, raising concern that Gal-3 may function primarily as a biomarker of renal dysfunction rather than a causal mediator of cardiovascular disease. Studies in heart failure populations often show strong correlations between Gal-3, congestion, and impaired kidney function, complicating the attribution of Gal-3 to cardiac pathology per se. However, several lines of evidence indicate that the association between Gal-3 and coronary atherosclerosis cannot be fully explained by renal confounding. First, prospective cohort analyses that adjust for eGFR, albuminuria, and cystatin-C consistently demonstrate an independent relationship between Gal-3 and incident CAD events. Second, experimental models show that Gal-3 produced by activated macrophages and stressed vascular cells drives fibro-inflammatory remodeling within the arterial wall, a mechanism distinct from the renocardiac axis observed in HF. Thus, although renal function influences circulating concentrations, Gal-3 reflects—and contributes to—vascular inflammation through pathways not attributable solely to impaired clearance. Together, these findings confirm that Gal-3 elevation in CAD cannot be dismissed as a mere surrogate of impaired renal clearance. Further evidence supporting a cardiorenal role for Gal-3 comes from the DECLARE–TIMI 58 biomarker substudy (*n* = 14,530). In this large cohort, higher baseline Gal-3 concentrations were strongly and independently associated with accelerated decline in renal function and greater risk of kidney outcomes. Importantly, the absolute renal benefit of dapagliflozin was largest in patients with the highest Gal-3 levels, suggesting that Gal-3 may identify a phenotype characterized by heightened fibro-inflammatory activity across both vascular and renal tissues. These findings reinforce that Gal-3 should be considered not only a vascular biomarker but a broader pan-fibrotic indicator with therapeutic implications [62]. It is also notable that Mendelian randomization (MR) analyses have not demonstrated a causal relationship between genetically elevated Gal-3 levels and incident CAD or heart failure. In a large MR study [63], variants associated with higher circulating Gal-3 did not increase cardiovascular risk, suggesting that Gal-3 functions primarily as a downstream mediator and biomarker of fibro-inflammatory activity rather than an upstream causal driver of disease. Importantly, Gal-3 represents a convergence point where lysosomal dysfunction, immune activation, and extracellular matrix remodeling intersect—mechanisms insufficiently targeted by conventional lipid-lowering or anti-inflammatory therapies [13,31,32,55,56,57,58,60]. These properties position Gal-3 as a mechanistic driver and biomarker of residual cardiovascular risk [13,14,30,41,50].

Clinical translation of Gal-3 inhibition has also begun to emerge. Belapectin (GR-MD-02), a Gal-3-targeting polysaccharide, demonstrated reductions in portal pressure and improvements in fibrosis-related endpoints in patients with NASH and cirrhosis [64], although effects were not uniform across subgroups. Modified citrus pectin (MCP), a competitive CRD inhibitor, has shown acceptable safety in early-phase studies; however, a randomized trial in hypertension did not demonstrate improvement in blood pressure despite adequate target engagement [65]. This negative result highlights two important considerations: (1) Gal-3 inhibition may exert organ-specific rather than systemic hemodynamic effects, and (2) appropriate patient selection—particularly identification of a fibro-inflammatory phenotype—may be essential for demonstrating therapeutic benefit. These early studies highlight that Gal-3 is pharmacologically targetable and biologically relevant, supporting continued investigation in cardiovascular settings.

Collectively, Gal-3 represents a pivotal mediator within the glyco-inflammatory axis, translating intracellular stress signals into chronic vascular inflammation and fibrosis [14,30,31,32,60]. The following section will explore how this molecular versatility contributes specifically to the initiation, progression, and clinical expression of coronary atherosclerosis.

## 4. Gal-3 in Coronary Atherosclerosis and Plaque Progression

Atherosclerosis is a chronic inflammatory process characterized by lipid deposition, immune activation, and ECM remodeling within the arterial wall [30,31,32]. Accumulating evidence implicates Gal-3 as a central effector molecule that integrates these processes across multiple stages of plaque evolution—from lesion initiation to rupture [13,18,30,32,41,60]. Importantly, increasing data indicate that these effects are not merely lectin-receptor interactions but reflect disease-associated glycan remodeling that enhances Gal-3 binding and lattice formation within the atherosclerotic microenvironment.

### 4.1. Expression and Localization in Atherosclerotic Lesions

Histopathological and immunohistochemical studies have demonstrated intense Gal-3 expression in macrophage-rich regions of human and experimental atherosclerotic plaques [32,60,66]. Gal-3-positive macrophages are concentrated at vulnerable plaque shoulder regions, where inflammatory activity and matrix degradation are prominent [33,66,67]. VSMCs and fibroblasts within the fibrous cap also express Gal-3, suggesting its dual role in both inflammatory amplification and fibrotic stabilization [30,31,32,60]. Moreover, endothelial cells overlying early fatty streaks express Gal-3 in response to disturbed flow and oxidized low-density lipoprotein, promoting monocyte adhesion through VCAM-1 and integrin up-regulation [25,61,68]. Recent studies additionally show that inflamed plaque regions undergo characteristic glycan remodeling—particularly increased β1,6-GlcNAc branching, poly-LacNAc extension, and α1-2 fucosylation—that markedly enhance Gal-3 binding avidity. This provides a mechanistic explanation for the preferential enrichment of Gal-3 at plaque shoulders and lipid-laden macrophage zones.

### 4.2. Mechanistic Insights from Experimental Models

**Figure 2:**
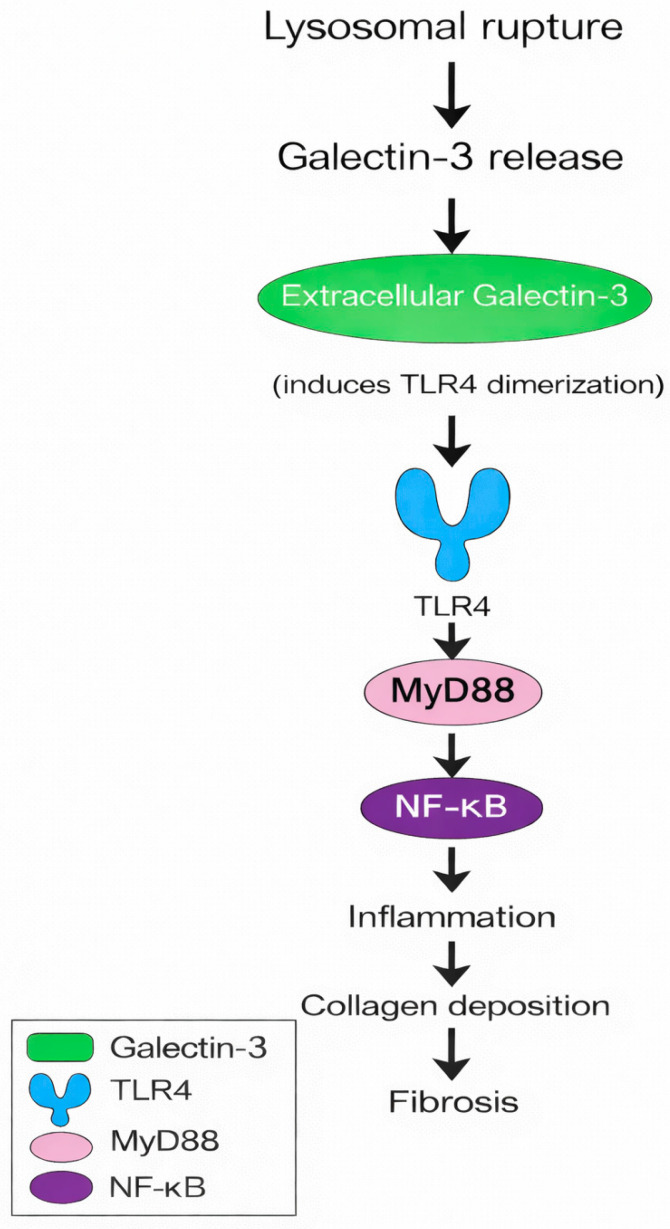
Extracellular Galectin-3 released from damaged lysosomes activates TLR4–MyD88–NF-κB signaling and promotes fibrosis. MyD88, Myeloid Differentiation Primary Response Protein 88; NF-κB, Nuclear Factor Kappa-Light-Chain-Enhancer of Activated B Cells; TLR4, Toll-Like Receptor 4.

In ApoE^−^/^−^ and LDLR^−^/^−^ mice, Gal-3 deficiency markedly reduces macrophage infiltration, plaque size, and necrotic core formation, accompanied by attenuated proinflammatory cytokine (IL-1β, TNF-α) and matrix metalloproteinase expression [32,34,36,60]. Conversely, exogenous Gal-3 administration enhances monocyte recruitment, foam-cell formation, and collagen deposition [35,37]. Thus, Gal-3 drives both the inflammatory and fibrotic atherogenesis arms [31,32,36,60]. Mechanistically, Gal-3 binds to glycosylated receptors such as TLR4 and TGF-βR on macrophages and VSMCs, thereby activating NF-κB and Smad-dependent pathways, perpetuating inflammation and matrix remodeling [38,39,69] (Figure 2). In vitro, Gal-3 stimulates the endothelial–mesenchymal transition and promotes reactive oxygen species production, linking oxidative stress to fibrotic remodeling [25,70]. Importantly, several in vivo studies demonstrate that these effects are glycan-dependent: disruption of the Gal-3–glycan lattice—either by genetic deletion of Gal-3 or by competitive CRD inhibition—attenuates inflammatory signaling and limits necrotic core expansion. These findings link disease-associated glycosylation patterns directly to the amplification of Gal-3-mediated plaque biology.

### 4.3. Clinical Correlations and Prognostic Value

In patients with stable CAD, serum Gal-3 levels correlate with plaque burden assessed by intravascular ultrasound and coronary computed tomography angiography [13,18,41,71]. Importantly, elevated Gal-3 has been associated with thin-cap fibroatheroma, a vulnerable plaque histological hallmark [33,66,67]. Furthermore, high circulating Gal-3 concentrations predict major adverse cardiovascular events (MACE) independent of traditional risk factors and LDL-C levels, underscoring its relevance to residual cardiovascular risk [13,14,30,41,50]. In ACS, Gal-3 levels rise rapidly following myocardial injury, remaining elevated during post-infarction remodeling, thereby reflecting ongoing inflammation and fibrosis [17,30,44]. These clinical associations may reflect systemic glycan remodeling characteristic of cardiometabolic stress—patterns that increase Gal-3 lattice formation and thereby amplify fibro-inflammatory signaling within plaques. Therefore, Gal-3 represents both a biomarker and a pathogenic mediator linking chronic inflammation to adverse remodeling in coronary disease [13,14,17,18,25,41].

### 4.4. Integrative Perspective

Collectively, Gal-3 acts as a molecular bridge connecting intracellular lysosomal stress with extracellular matrix remodeling and immune activation, a concept increasingly recognized in recent cardiovascular lysosomal biology frameworks [25,31,32,40,55,57]. Its simultaneous involvement in macrophage recruitment, VSMC activation, and collagen synthesis explains its paradoxical association with both plaque vulnerability and reparative fibrosis [31,32,34,36,37,60]. Importantly, this dual nature positions Gal-3 at the crossroads of residual inflammatory and fibrotic risk, processes inadequately controlled by conventional lipid-lowering or anti-cytokine therapies [13,14,17,25,41,50]. Comparable limitations have been observed in plaque regression trials, where residual risk persists despite maximal LDL-C lowering [10]. Thus, targeting the Gal-3–glycan–inflammation axis may offer a novel therapeutic strategy to attenuate residual risk and promote plaque stabilization, addressing disease mechanisms not captured by current cardiometabolic therapies.

## 5. Clinical Implications: Gal-3 as a Biomarker and Therapeutic Target

### 5.1. Gal-3 Inhibitors: Mechanistic Basis of Glycan-Dependent Blockade

Gal-3 inhibitors function by disrupting the carbohydrate-mediated interactions that enable Gal-3 lattice formation and sustained proinflammatory signaling. Since Gal-3 requires multivalent engagement with poly-LacNAc-extended, β1,6-GlcNAc-branched N-glycans to oligomerize, effective inhibitors act through one of three mechanistic strategies: (1) competitive blockade of the carbohydrate-recognition domain (CRD), preventing Gal-3 from binding LacNAc motifs; (2) disruption of N-terminal oligomerization, inhibiting the assembly of supramolecular lattices; or (3) masking or enzymatically modifying Gal-3-permissive glycans on target receptors. MCP and its derivative GR-MD-02 inhibit Galectin-3 by competitively binding to the CRD. These agents are enriched in low-molecular-weight, galactose-containing pectic fragments that mimic endogenous β-galactoside-containing glycans, thereby occupying the CRD and preventing Gal-3 engagement with poly-N-acetyllactosamine-extended N-glycans. Biochemical and biophysical studies, including competitive binding assays and surface plasmon resonance analyses, have demonstrated direct interaction between these galactose-rich polysaccharide fragments and the Gal-3 CRD in vitro, resulting in impaired lattice formation and reduced receptor clustering [72,73]. Novel small-molecule inhibitors similarly occupy the CRD pocket, preventing Gal-3 from stabilizing TGF-β receptor and integrin signaling complexes. By dismantling or preventing Gal-3 lattices, these agents interrupt the sustained SMAD, NF-κB, and MAPK activation that drives fibrosis and chronic vascular inflammation.

### 5.2. Gal-3 as a Residual Cardiovascular Risk Biomarker

Despite aggressive lipid-lowering therapy, several patients with CAD continue to experience cardiovascular events, reflecting persistent residual inflammatory and fibrotic risk [14,25,50]. Gal-3 has emerged as a promising biomarker that captures these non-lipid pathways of disease progression [14,25,31,32]. Elevated plasma Gal-3 levels are consistently observed in individuals with established CAD, heart failure, and metabolic syndrome [13,17,18,25,41]. Unlike traditional markers such as CRP, Gal-3 reflects both systemic inflammation and local fibro-inflammatory activity within the vascular wall and myocardium [31,32,34,36,60,74]. Within the broader cardiovascular biomarker framework, Gal-3 is classified as a remodeling- and fibrosis-associated biomarker, together with ST2 and GDF-15, distinguishing it from injury markers (e.g., troponins) and hemodynamic stress markers (e.g., B-type natriuretic peptide [BNP]/N-terminal pro-B-type natriuretic peptide), thereby highlighting that Gal-3 reflects chronic macrophage-driven tissue remodeling rather than acute cardiomyocyte injury, aligning it with long-term residual risk rather than short-term hemodynamic instability [75]. Cross-sectional and longitudinal studies demonstrate that high Gal-3 concentrations predict plaque instability, myocardial remodeling, and adverse outcomes independently of LDL-cholesterol, high-sensitivity CRP, and renal function [13,14,17,41]. Thus, Gal-3 measurement provides additive prognostic information for identifying patients at high residual risk beyond conventional biomarkers [1,13,14,41].

### 5.3. Predictive and Prognostic Value in Clinical Settings

Several large-scale clinical studies have evaluated the prognostic performance of Gal-3 across the cardiovascular continuum (Table 1). In stable CAD, elevated baseline Gal-3 levels are associated with greater coronary plaque burden and increased risk of MACE over long-term follow-up [13,41]. In ACS, Gal-3 levels rise rapidly following myocardial infarction, remaining persistently elevated, correlating with infarct size, left-ventricular remodeling, and subsequent heart-failure development [17,44,76]. Meta-analyses have confirmed that patients in the highest Gal-3 quartile exhibit a two- to three-fold increased cardiovascular mortality risk, even after conventional risk factor and natriuretic peptide adjustment [14]. Beyond cardiovascular outcomes, elevated Gal-3 predicts accelerated renal function decline and increased cardiorenal event risk in patients with diabetes and metabolic syndrome, reflecting its role as a mediator of the heart–kidney–metabolic axis. Therefore, Gal-3 is not confined to cardiac remodeling; instead, it reflects systemic fibro-inflammatory remodeling across multiple organs [77]. Similarly, Gal-3 integrates multiple pathophysiologic dimensions—immune activation, fibrosis, and metabolic dysregulation—thereby functioning as a global index of cardiovascular vulnerability [30].

### 5.4. Gal-3 Axis Therapeutic Targeting

Given its central role in the glyco-inflammatory network, Gal-3 represents an attractive therapeutic target for mitigating residual risk (Table 2). Interventional evidence further supports its causal role in vascular pathology: pharmacological inhibition attenuates macrophage-driven inflammation and vascular fibrosis, leading to significantly reduced atherosclerotic progression. Thus, in CAD, Gal-3 is not merely a biomarker but a pathogenic mediator [78]. Accordingly, recent mechanistic reviews highlight Gal-3 as a central integrator of inflammation, fibrosis, and metabolic dysregulation across cardiovascular disease phenotypes, further reinforcing its suitability as a therapeutic node [30].

Experimental Gal-3 inhibition using small molecules (e.g., TD139, GB1107), modified citrus pectin, or antisense oligonucleotides has demonstrated anti-inflammatory and anti-fibrotic effects in preclinical atherosclerosis and cardiac remodeling models [36,79,80].

In models of diabetic myocardial injury, Gal-3 inhibition with modified citrus pectin has been demonstrated to reduce cardiac inflammation, oxidative stress, and fibrotic remodeling, preserving left ventricular function following isoprenaline-induced myocardial infarction in type 2 diabetes. Thus, Gal-3 represents a mechanistic driver of cardio-metabolic myocardial remodeling, further supporting its therapeutic tractability [81]. Importantly, Gal-3 is both prognostic and therapeutically modifiable.

In patients undergoing lipoprotein apheresis for drug-refractory hypercholesterolemia, plasma Gal-3 concentrations were reduced by approximately 20%, regardless of LDL-C lowering [82]. Therefore, Gal-3 represents a dynamic effector molecule rather than a static biomarker, reinforcing its potential as a therapeutic target. These interventions reduce macrophage activation, suppress TGF-β/Smad signaling, and limit extracellular matrix deposition [31,74,83]. Early-phase clinical trials of inhaled TD139 in pulmonary fibrosis have demonstrated safety and target engagement, supporting Gal-3 inhibition translational feasibility [84,85]. Although cardiovascular-specific trials remain scarce, the mechanistic rationale suggests that modulating Gal-3 activity may complement lipid-lowering and anti-cytokine therapies to achieve more complete vascular protection [14,30].

### 5.5. Integration into Precision Cardiovascular Medicine

Incorporating Gal-3 into multimarker panels could refine risk stratification and therapeutic decision-making in CAD [14,86]. Combined assessment of Gal-3 with inflammatory markers (CRP, IL-6), fibrotic markers (TGF-β, procollagen peptides), and metabolic indicators (glycated hemoglobin, adiponectin) may help identify patients who would benefit from anti-fibrotic or immunomodulatory strategies [8,16,30]. Moreover, serial Gal-3 monitoring could represent a dynamic tool to track treatment response or detect early relapse of vascular inflammation [42,43,45]. Ultimately, targeting the Gal-3-mediated glyco-inflammatory axis represents a novel avenue to reduce residual cardiovascular risk in the post-statin era [1,30,41]. Overall, Gal-3 should be regarded both as a biomarker of disease burden and as a mechanistic driver of fibro-inflammatory residual cardiovascular risk, representing a rational and actionable therapeutic target in precision cardiovascular medicine.

## 6. Future Perspectives and Conclusions

The concept of residual cardiovascular risk has reshaped the landscape of CAD research, emphasizing that lipid-lowering alone cannot fully suppress the complex biological processes driving atherothrombosis [1,14,30]. Increasing evidence positions the glyco-inflammatory axis, particularly Gal-3, at the intersection of metabolic stress, immune activation, and tissue remodeling—key determinants of this residual risk [15,32,83]. Understanding and modulating this axis will be critical for the next generation of cardiovascular prevention and therapy approaches.

### 6.1. Future Research Directions

Advances in glycomics, proteomics, and metabolomics enable comprehensive profiling of glycosylation changes and lectin–glycan interactions in cardiovascular disease [23,87,88,89,90,91,92]. Integrating these omics technologies with single-cell transcriptomics and spatial proteomics could uncover cell-specific glycan signatures dictating immune and fibrotic responses within atherosclerotic plaques [93,94]. Furthermore, elucidating how metabolic disorders, including diabetes and obesity, reprogram glycosylation pathways may clarify inter-individual variations in Gal-3 activity and clinical outcomes [24,25,27]. These approaches will facilitate the identification of novel biomarkers and therapeutic nodes along the glyco-inflammatory cascade [30,36,74,76,79,80].

### 6.2. Translational and Therapeutic Perspectives

Targeting Gal-3 and related glycan–lectin pathways offers a unique opportunity to address both inflammatory and fibrotic residual risk components [13,32,83]. This therapeutic rationale is consistent with recent advances highlighting lysosomal stress as a key upstream trigger of Gal-3-mediated inflammatory and fibrotic signaling in cardiovascular disease [40]. Small-molecule inhibitors and glycomimetic agents are being refined for improved selectivity and bioavailability [36,79,80].

**Figure 3:**
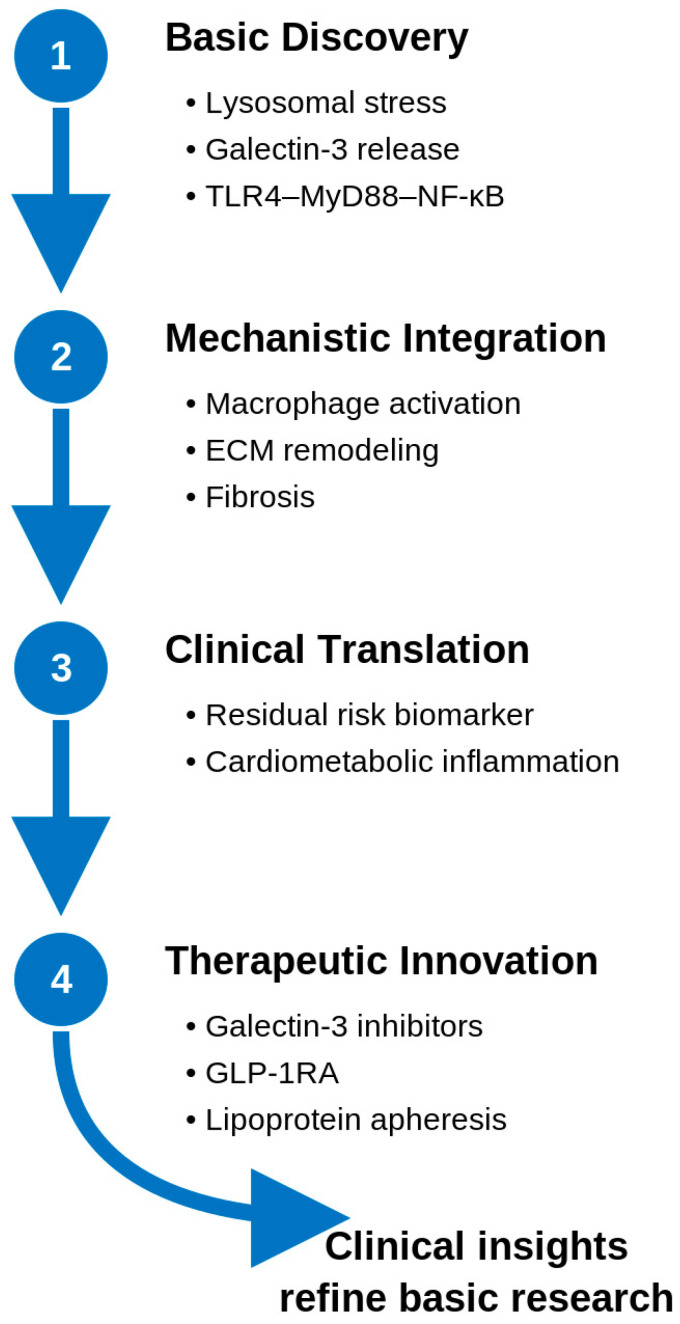
Translational continuum from basic discovery of lysosomal stress and Galectin-3 release to therapeutic innovation targeting the glyco-inflammatory axis. ECM, Extracellular matrix; GLP-1RA, glucagon-like peptide-1 receptor agonist; MyD88, Myeloid Differentiation Primary Response Protein 88; NF-κB, Nuclear Factor Kappa-Light-Chain-Enhancer of Activated B Cells; TLR4, Toll-Like Receptor 4.

Beyond direct Gal-3 inhibition, incretin-based therapies such as GLP-1 receptor agonists have demonstrated preliminary evidence of modulating Gal-3 expression indirectly. For instance, in a Gubra Amylin nonalcoholic steatohepatitis diet-induced nonalcoholic steatohepatitis mouse model, semaglutide reduced hepatic Gal-3 expression and improved fibrosis and inflammation [95]. Although clinical studies in obesity and diabetes have reported minimal or variable circulating Gal-3 changes, these agents may exert context-dependent effects through metabolic and immunomodulatory pathways (Table 3). The broader translational framework linking these mechanistic insights to therapeutic innovation is illustrated in Figure 3.

Combining Gal-3 inhibition with established therapies, such as SGLT2 inhibitors, GLP-1 receptor agonists, or anti-cytokine biologics, may yield synergistic benefits by simultaneously modulating metabolic, inflammatory, and fibrotic axes [16,96]. In parallel, circulating Gal-3 could be integrated into personalized monitoring algorithms, allowing dynamic assessment of vascular inflammation and therapeutic response [14,45].

**Table 3:** Gal-3 modulation by incretin-based or metabolic therapies.

Drug/Class	Model or Trial	Target (Sample)	Direction of Gal-3 Change	Key Findings	Reference
Semaglutide (GLP-1RA)	GAN diet-induced NASH mouse model	Hepatic tissue Gal-3 (immunostaining)	↓ Decrease	Semaglutide reduced hepatic Gal-3 expression and fibrosis with improved inflammation.	Hansen et al. [97]
Liraglutide/Dual incretin (GLP-1/GCG)	Diet-induced obesity/NASH mice	Hepatic Gal-3 (macrophage marker)	↓ Decrease	Reduced Gal-3-positive Kupffer and stellate cells, attenuating fibrotic remodeling.	Perakakis et al. [98]
Liraglutide (GLP-1RA)	Randomized clinical trial in obesity/prediabetes	Circulating (plasma) Gal-3	No change	Despite weight loss comparable to lifestyle intervention, plasma Gal-3 remained unchanged.	Simeone et al. [99]
Dapagliflozin (SGLT2i)	DECLARE–TIMI 58 biomarker substudy (T2DM)	Baseline plasma Gal-3	Predictive, not reduced	Higher baseline Gal-3 identified patients with greater absolute risk reduction in kidney outcomes.	Haller PM et al. [62]

DECLARE–TIMI, Dapagliflozin Effect on Cardiovascular Events–Thrombolysis in Myocardial Infarction; Gal-3, Galectin-3; GAN, Gubra Amylin NASH; GCG, Glucagon; GLP-1, Glucagon-Like Peptide-1; GLP-1RA, Glucagon-Like Peptide-1 Receptor Agonist; NASH, Nonalcoholic Steatohepatitis; SGLT2i, Sodium–Glucose Cotransporter 2 Inhibitor; T2DM, Type 2 Diabetes Mellitus; ↓, decrease.

#### 6.2.1. Predictive Value of Gal-3 for Therapeutic Response

Beyond its role as a biomarker of disease activity, Gal-3 may represent a predictive marker of therapeutic responsiveness. In a recent post hoc analysis of the DECLARE-TIMI 58 trial, patients with higher baseline Gal-3 levels derived greater absolute risk reduction from SGLT2 inhibitor therapy, despite no consistent Gal-3 lowering. Therefore, Gal-3 may reflect an active fibro-inflammatory milieu that is particularly amenable to modulation by agents targeting metabolic and inflammatory stress pathways. This concept aligns with recent clinical data showing that SGLT2 inhibitors exert anti-inflammatory and redox-modulating effects in patients with type 2 diabetes [100,101,102]. Accordingly, Gal-3 might help identify subgroups that benefit most from SGLT2 inhibitors or other anti-fibrotic interventions, underscoring its value as a therapeutic stratification biomarker rather than a passive disease marker [62].

#### 6.2.2. Clinical Implementation and Future Therapeutic Strategies

Integrating Gal-3 into clinical practice will require standardized assays, reference ranges, and longitudinal validation across diverse patient populations. Moreover, its incorporation into multi-marker panels—alongside inflammatory, metabolic, and fibrotic indices—could refine patient stratification for anti-inflammatory and anti-fibrotic therapies. Furthermore, clinical trials targeting the Gal-3 axis, either through direct inhibition or combination strategies with incretin-based or lipid-modifying agents, should evaluate both biochemical and imaging-based endpoints to assess vascular remodeling. Such approaches may help translate mechanistic insights from experimental models into personalized interventions addressing residual cardiovascular risk more comprehensively.

### 6.3. Concluding Remarks

Gal-3 exemplifies how a single glycan-binding protein can bridge intracellular stress responses with extracellular inflammatory and fibrotic remodeling, embodying a mechanistic “missing link” between lysosomal dysfunction, immune activation, and matrix deposition—pathways that collectively sustain residual cardiovascular risk even in optimally treated patients. Incorporating the glyco-inflammatory paradigm into cardiovascular research broadens our mechanistic understanding and opens new intervention avenues. Future clinical studies targeting Gal-3 or its downstream signaling networks may herald a paradigm shift toward precision cardio-metabolic medicine, where glycosylation and lectin biology modulation become an integral strategy for achieving comprehensive vascular protection.

## Figures and Tables

**Table 1:** Summary of key clinical studies evaluating Gal-3 in CAD and ACS.

Study (First Author, Year)	Population/Design	Endpoint(s)	Main Findings	Ref. No.
Obeid S. et al., 2020	295 patients with ACS	Gal-3 vs. MACE and LV function at 6 months	Gal-3 levels at admission predicted MACE and adverse LV remodeling independent of NT-proBNP.	[17]
Choi Y.J.; Seo S.M., 2025	418 patients undergoing PCI with drug-eluting stents	12-month MACE composite	Elevated baseline Gal-3 independently predicted recurrent events and stent restenosis after PCI.	[41]
Banerjee S. et al., 2025 (meta-analysis)	19 cohorts >14,000 participants with CVD or CKD	CV and all-cause mortality	Highest Gal-3 quartile → ≈ 2.4-fold higher CV mortality after adjustment for traditional factors.	[14]
Li M. et al., 2022	260 patients with stable CAD	Coronary stenosis severity and Gal-3 levels	Serum Gal-3 positively correlated with stenosis severity and plaque instability.	[44]
Di Gregoli K. et al., 2020	Human atherosclerotic plaques (vulnerable vs. stable)	Immunohistochemical localization of Gal-3 and macrophage markers	Gal-3 intensely expressed in macrophage-rich shoulder regions of vulnerable plaques; co-localized with MMP-9 and oxidized lipids.	[33]

ACS, Acute Coronary Syndrome; CAD, Coronary Artery Disease; CKD, Chronic Kidney Disease; CV, Cardiovascular; Gal-3, Galectin-3; LV, Left Ventricular; MACE, Major Adverse Cardiovascular Events; MMP-9, Matrix Metalloproteinase-9; NT-proBNP, N-terminal pro-B-type Natriuretic Peptide; PCI, Percutaneous Coronary Intervention.

**Table 2:** Therapeutic strategies directly or indirectly targeting the Gal-3 axis.

Intervention/Class	Mechanism of Action	Evidence Stage	Key Outcomes
TD139 (GB0139)	CRD blockade (direct Gal-3 inhibition)	Phase 1b–2a (IPF/COVID pneumonitis)	Safe, clear target engagement
GB1107	Small-molecule Gal-3 antagonist	Preclinical	↓ inflammation, ↓ fibrosis in vascular/cardiac models
Modified Citrus Pectin	Competitive binding to Gal-3 carbohydrate sites	Preclinical/Early clinical supplement use	↓ macrophage activation, ↓ fibroblast activation
Lipoprotein Apheresis	Removes circulating Gal-3 along with LDL and inflammatory mediators	Clinical use in dyslipidemia (J Clin Apher 2016)	~20% Gal-3 reduction per session; supports “modifiability”
Antisense or siRNA-based Gal-3 knockdown (experimental)	Transcriptional suppression	Preclinical	Strong anti-fibrotic + anti-inflammatory effects

CRD, Carbohydrate Recognition Domain; Gal-3, Galectin-3; IPF, Idiopathic Pulmonary Fibrosis; LDL, Low-Density Lipoprotein; siRNA, Small Interfering RNA; ↓, decrease.

## Data Availability

No new data were created or analyzed in this study.

## Abbreviations

The following abbreviations are used in this.

ACS	Acute Coronary Syndrome
BNP	B-Type Natriuretic Peptide
CAD	Coronary Artery Disease
CKD	Chronic Kidney Disease
CRD	Carbohydrate Recognition Domain
CRP	C-Reactive Protein
CV	Cardiovascular
DECLARE–TIMI	Dapagliflozin Effect on Cardiovascular Events–Thrombolysis in Myocardial Infarction
ECM	Extracellular Matrix
GCG	Glucagon
GDF-15	Growth Differentiation Factor-15
GLP-1	Glucagon-Like Peptide-1
GLP-1RA	Glucagon-Like Peptide-1 Receptor Agonist
IL-1β	Interleukin-1 Beta
IL-6	Interleukin-6
IPF	Idiopathic Pulmonary Fibrosis
LDL	Low-Density Lipoprotein
LDL-C	Low-Density Lipoprotein Cholesterol
LDLR^−^/^−^	Low-Density Lipoprotein Receptor Knockout
LMP	Lysosomal Membrane Permeabilization
LV	Left Ventricular
MACE	Major Adverse Cardiovascular Events
MMP-9	Matrix Metalloproteinase-9
MyD88	Myeloid Differentiation Primary Response Protein 88
NASH	Nonalcoholic Steatohepatitis
NF-κB	Nuclear Factor Kappa-Light-Chain-Enhancer of Activated B Cells
NT-proBNP	N-Terminal pro–B-Type Natriuretic Peptide
PCI	Percutaneous Coronary Intervention
siRNA	Small Interfering RNA
SGLT2i	Sodium–Glucose Cotransporter 2 Inhibitor
T2DM	Type 2 Diabetes Mellitus
TGF-β	Transforming Growth Factor-Beta
TLR4	Toll-Like Receptor 4
TNF-α	Tumor Necrosis Factor-Alpha
VSMC	Vascular Smooth-Muscle Cell
